# Three Dimensional Hybrids of Vertical Graphene-nanosheet Sandwiched by Ag-nanoparticles for Enhanced Surface Selectively Catalytic Reactions

**DOI:** 10.1038/srep16019

**Published:** 2015-11-02

**Authors:** Jing Zhao, Mentao Sun, Zhe Liu, Baogang Quan, Changzhi Gu, Junjie Li

**Affiliations:** 1 Beijing National Laboratory for Condensed Matter Physics, Institute of Physics, Chinese Academy of Sciences, P. O. Box 603, Beijing 100190, China

## Abstract

Three dimensional (3D) plasmonic nanostructure is perfect for the surface-enhanced Raman scattering (SERS) and also very suitable for surface catalytic reaction, but how to design and fabricate is still a robust task. Here, we show a 3D plasmonic nanohybrid of vertical graphene-nanosheet sandwiched by Ag-nanoparticles on the silicon nanocone array substrate for enhanced surface catalytic reaction. By SERS detection, we find that this hierarchical nanohybrid structure is highly efficient in the enhancement of catalytic reaction, even at a very low concentration of 10^−11^ M, which is far better than previous reports by four orders of magnitude. A strong electric field enhancement produced in the 3D framework nanohybrids of graphene nanosheet/Ag-nanoparticles is responsible for this great enhancement of catalytic reaction, due to larger electron collective oscillation in the composite system. Especially the oxygen adsorbed on the graphene and Ag nanoparticles can be excited to triplet excited states, and the electrons on the graphene and the nanoparticles can be effectively transferred to the oxygen, which plays very important role in molecular catalytic reactions. Our results demonstrate the contribution of graphene in plasmon-driven catalytic reactions, revealing a co-driven reaction process.This excellent SERS substrate can be used for future plasmon and graphene co-catalytic surface catalytic reactions, graphene-based surface plasmon sensors and so on.

Noble metallic nanoparticle catalysts and their use in catalytic processes have recently been widely investigated experimentally and theoretically due to their advantages in nanoscience and nanotechnology. Since the first report of plasmon-driven catalytic reaction of p-aminothiophenol (PATP) oxidizing to dimercaptoazobenzene (DMAB) revealed by surface-enhanced Raman scattering (SERS)^1^,^2^, many efforts have been made to confirm the occurrence of this catalytic reaction and explore mechanisms of plasmon-induced catalytic reactions^3^,^4^,^5^,^6^,^7^,^8^,^9^,^10^. Subsequently, the plasmon-driven catalytic reaction of the 4-nitrobenzenethiol (4NBT) reducing to DMAB has also been widely investigated^11^,^12^,^13^,^14^,^15^,^16^. It has been found that surface plasmon resonance can activate the oxygen to the triplet excited state, which plays very important role in the plasmon-driven catalytic reactions^10^. Now, surface plasmon-driven catalytic reaction is becoming a booming field, which is called plasmon-chemistry^17^,^18^,^19^,^20^,^21^ Direct plasmon-driven catalytic reaction has great advantages compared with conventional catalytic reaction, as summarized in a recent review^22^.

Graphene, as an atomic-scale honeycomb lattice made of carbon atoms, conducts heat and electricity with great efficiency. Recently, graphene has also been used as the substrate of SERS^16^,^23^,^24^,^25^,^26^, as the evidence for chemical mechanism of SERS enhancement, wherein electrons transfer between graphene and the target molecules. Graphene has also drawn extensive attention in the field of semiconductor photocatalysis due to its excellent electronic structure and electronic transmission performance. However, the study of graphene in the field of direct plasmon-driven catalytic reaction is little^27^,^28^,^29^, and the mechanism and the role of graphene in plasmon-driven catalytic reactions are unclear. For example, can photoexcited graphene activate the oxygen to the triplet excited state for catalytic reactions? And how is the hybrid system of graphene enhancing plasmon-driven toward catalytic reaction? Therefore, it is a great challenge to reveal the mechanism behind the question and to design a practical graphene-based nanostructure for a highly efficient catalytic reaction.

Recently, the graphene-nanoparticle hybrids were reported to be able to tune the enhancement of Raman scattering^30^. The as-reported graphene-nanoparticle hybrids are essentially two dimensional (2D) system, in which a single layer graphene (SLG) is usually used to compound with noble metallic nanoparticles so that some limitations are formed, such as frangible surface, complex transferring, low surface-to-volume and only single sided composite with nanoparticles, etc.^31^,^32^ Therefore, for SERS, or surface catalytic reaction substrate, 3D hybrids of graphene-nanoparticle nanostructure should be much better selection than 2D hybrids because it has a three-dimensional space with free spatial orientation, high surface-to-volume and two-sided composite with nanoparticles that can bring high density, high intensity plasmonic coupling effect^33^,^34^. In this approach, the vertical few-layer greaphene nanosheet are an ideal candidate for 3D graphene-nanoparticle hybrids, which can be easily grown by chemical vapor deposition with a large area and controllable morphology.

In this work, we fabricated a 3D hierarchical nanostructure of vertical flower-like graphene nanosheets (FGNSs) sandwiched by Ag nanoparticles (Ag-NPs) grown on silicon nanocone arrays substrate, in which silicon nanocone arrays can further optimize the 3D hierarchical hybrid structure and greatly improve its surface-to-volume. Firstly we verified that this hierarchical hybrid structures have a great SERS ability to be a perfect SERS substrate. Further, to demonstrate that this excellent SERS substrate is very suitable for graphene enhancing plasmon-driven catalytic reactions, PATP and 4NBT were separately oxidized and reduced to DMAB. Our results demonstrate that this hierarchical hybrid structure is very efficient in graphene enhancing plasmon-driven catalytic reaction, even at the level of 10^−11^ M. A mechanism of graphene enhancing plasmon-driven catalytic reaction is proposed and discussed here. This excellent 3D SERS substrate can be used for future graphene and plasmon co-driven surface catalytic reactions, graphene-based surface plasmon sensors and so on.

## Results and Discussion

### D Nanostructure of Graphene-nanosheet/Ag-Nanoparticle Hybrids

**Figure 1:**
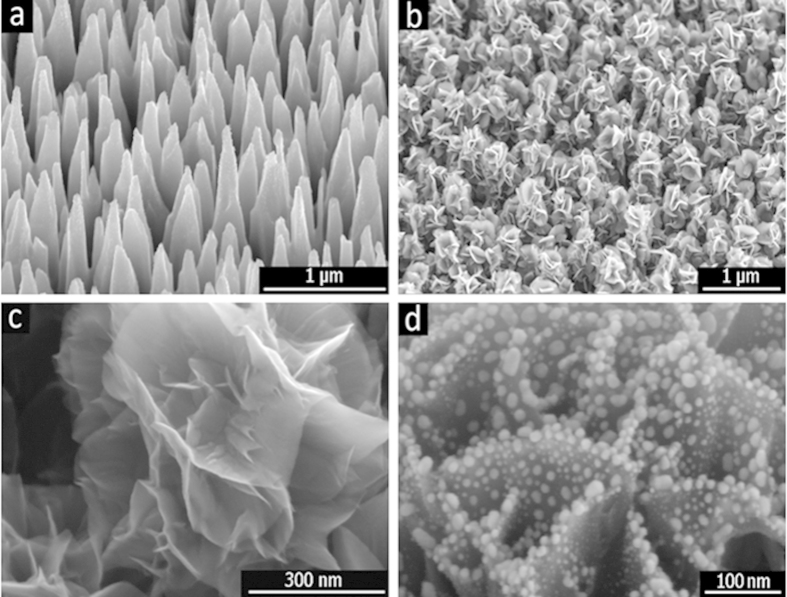
SEM images of (**a**) silicon nanocone array, which were fabricated by the maskless etching in the ICP system, (**b**) the floral-clustered graphene nanosheets, which were grown on the nanocone array with the growth times of 30 minutes, (**c**) the high-resolution image of petaliform graphene nanosheets on the nanocone tips, (d) Ag nanoparticles attached to both sides of a graphene nanosheet.

Figure 1a shows an SEM image, which is silicone nanocone array that were fabricated by inductively coupled plasma etching (see Experimental Section), which is used as the substrate for growing vertical flower-like graphene nanosheets (FGNSs). As-etched cones are approximately 1.3 μm in the height with the tip radius of less than 30 nm, and the distribution density is around 2.5 × 10^9^/cm^2^. The FGNSs were uniformly grown on the silicon nanocone array by hot filament chemical vapor deposition using the mixture of H_2_ and CH_4_. Figure 1b shows the morphology of graphene nanosheets that were grown on the silicon nanocone arrays. By taking advantage of silicon conical nanostructures, few-layer graphene can be grown on it into nanosheets that are more fully bloomed and better dispersed than those synthesized on a planar surface. These graphene nanosheets were entirely covered on the cone surface, which is resemble the shapes of the flower petals. The FGNS clusters were isolated from each other, because of the conical array’s structure, which can effectively optimizes the density of FGNS arrays. The high-resolution SEM image (demonatrates in Fig. 1c) shows a clear view of edges of the FGNS on theses cone tip, moreover their petaliform edges were very thin, and spreading out layer by layer. Silver nanoparticles are deposited on FGNSs by e-beam deposition with accurate thickness control and a rotating process, and the resulting Ag nanoparticles are diffused around both sides of graphene nanosheets to form the sandwiched nanostructure, as shown in Fig. 1d. Furthermore, the abundance of vertically aligned sharp, and very suitably separated the edges of few-layer graphene increase the local electrical field^35^.

**Figure 2:**
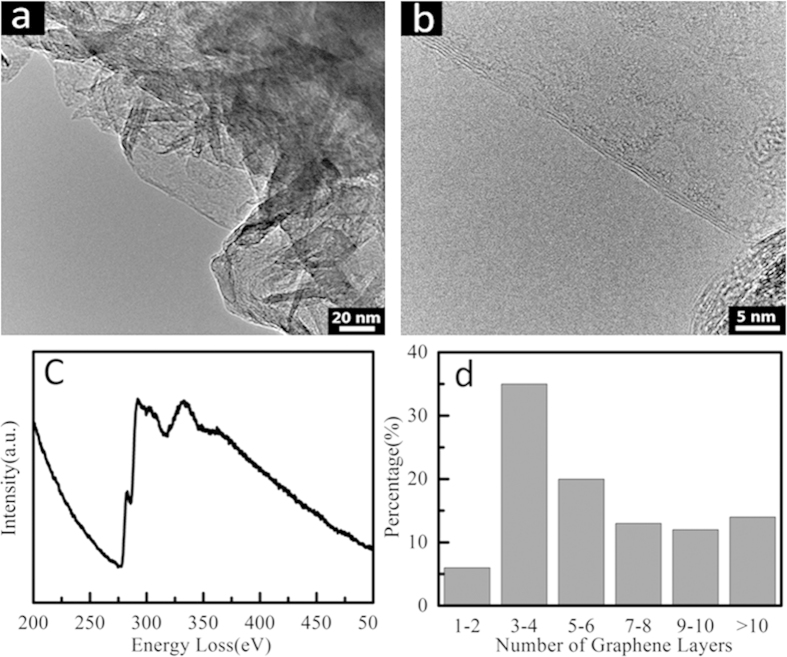
(**a**) The low-magnification TEM image of a single graphene sheet grown vertically on the surface of a silicon nanocone, (**b**) the HRTEM images of perfect FGNSs, (**c**) the typical EELS spectrum of FGNSs, and (**d**) Statistics of thickness distribution of FGNSs.

Furthermore, the structural details of the as-grown graphene nanosheets are acquired from TEM images and EELS, respectively, as shown in Fig. 2. Figure 2a is the TEM image of graphene fragments scraped from a FGNS, demonstrating the very thin sheet structures of graphene. Figure 2b is the HRTEM image of the FGNS, which clearly shows details of the few-layer graphene nanosheet. Furthermore, interference fringe of graphene edge shows that there are four layers graphene in the nanosheet, moreover, the interlayer spacing is 0.36 nm, which is larger than that of typical bulk graphite (0.34 nm), and implying a reduced van der Waals interaction. The electron energy loss spectroscopy (in Fig. 2c) revealed that bonding energy is about 320 eV, which is corresponding to the C–C bond of graphene. Statistics of the thickness distribution of FGNSs can be seen from Fig. 2d, which demonstrate that graphene nanosheets with 3–4 layers are dominant in our products.

### Plasmon-driven Catalytic Reactions enhanced by Graphene-Nanoparticle Hybrids

The Ag nanoparticles are coated on both sides of graphene nanosheet grown on the Si nanocone arrays, and the distribution of Ag nanoparticles can be tuned by Ag nanofilm with different thicknesses from 2 to 8 nm, in which 6 nm thick Ag nanofilm with 30 nm in average particle diameter is selected as next tested sample because this thickness exhibits the best SERS ability among different Ag thicknesses (see Fig. 1S in supporting information), which is very favorable to the next catalytic reaction detected by SERS.

**Figure 3:**
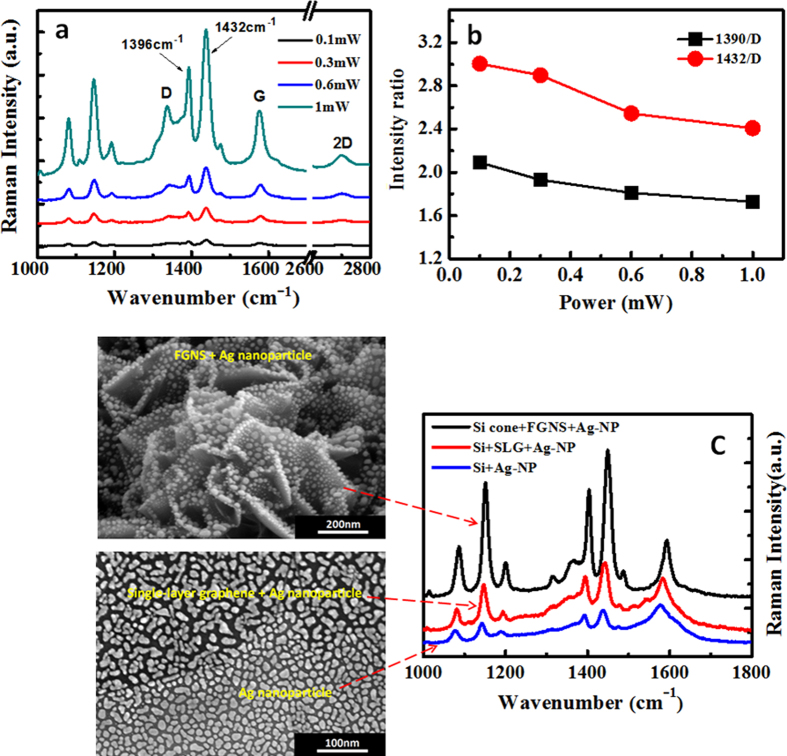
Plasmon-driven oxidized catalytic reaction. (**a**) PATP oxidized to DMAB at different laser powers by SERS; (**b**) the ratio of molecular Raman intensities over Raman intensity of graphene based on (**a**); (**c**) the schematic of three different substrates (left side) corresponding to their SERS spectra (right side) that indicates the effect of graphene on the SERS and catalytic surface catalytic reaction processes.

Figure 3a shows a Raman signal of the laser power dependent plasmon and graphene co-driven catalytic reaction for the 3D Ag nanoparticles supported on graphene coated silicon nanocone array, where PATP is oxidized to DMAB. The Raman spectrum of DMAB productions shows itself characteristic peaks with the based-peaks of graphene. The original intrinsic Raman spectra of graphene, PATP and DMAB productions are shown in Fig. 2S(a), which are used as a comparison. It is found that with the increase of laser power, the probability of plasmon and graphene co-driven catalytic reactions is almost the same, by comparing Raman peaks of produced DMAB at 1396 and 1432 cm^−1^ with the Raman D peak of graphene at 1335 cm^−1^ (see Fig. 3b), meanwhile two characteristic peaks of DMAB almost cover the D peak of graphene. This result reveals that with very weak laser power, the PATP can almost completely be oxidized to DMAB, which is consistent with our previous experimental studies^35^. In such a catalytic reaction, it is easy to observe the product, but it is not easy to observe the reactants, since this oxidized reactant is very sensitive to plasmon intensity, and very weak laser power can make such a catalytic reaction occur^36^. So we can conclude that the efficiency of graphene enhancing plasmon-driven catalytic reaction is very high, even at very weak plasmon intensity.

**Figure 4:**
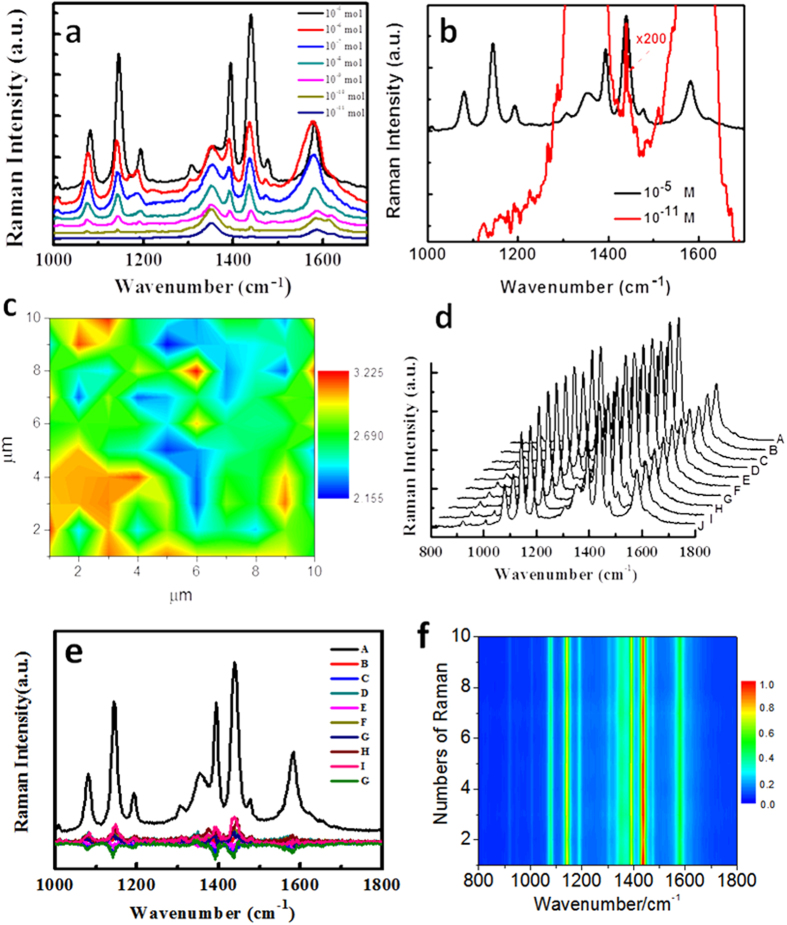
Molecular concentration dependent SERS of graphene enhancing plasmon-driven catalytic reaction and the uniform measurement of catalytic reaction at the laser intensity of 1 mW. (**a**) Molecular concentration dependent SERS of plasmon-driven catalytic reaction, (**b**) SERS spectra with ×200 at the concentration of 10^−11^, and (**c**) the uniform measurement of catalytic reaction. (**d**) The repeatability of SERS spectra by as-measured ten SERS spectra at intervals, (**e**) the difference of nine other SERS spectra, compared with SERS spectrum A in (**d**,**f** ) the 2D SERS spectra of (**d**).

In order to further demonstrate the significant effect of graphene nanosheets on this co-driven catalytic reaction, the SERS spectra of three kinds of substrate deposited by Ag nanoparticles using the same Ag film thickness of 6 nm – a pure planar silicon substrate, a CVD single-layer graphene (SLG) coated planar silicon substrate by transferring process and a silicon nanocone array coated by FGNSs – were measured under the same conditions for PATP, as shown in Fig. 3c. We can find that these three substrates all produce the DMAB with obvious characteristic peaks at 1396 and 1432 cm^−1^, but there are still huge differences. For the two produced peaks of DMAB, Ag nanoparticles deposited on the pure silicon substrate display a weaker intensity than those of the other two substrates. Silver nanoparticles deposited on a single-layer graphene coated silicon substrate show an obvious enhancement in the Raman peaks of DMAB compared with Ag nanoparticles on the pure silicon substrate, which give a direct proof that graphene can efficiently enhance plasmon-driven catalytic reaction. This result also indicates that as-transferred CVD graphene under Ag nanoparticles plays a key role in producing DMAB and is responsible for this Raman enhancement, because the addition of the CVD single layer graphene on the planar silicon wafer is the only difference between the two substrates. In particular, Ag nanoparticles deposited on flower-like graphene nanosheets, which were grown on the silicon nanocone array show more significant enhancement in the SERS intensity than planar silicon substrate coated by CVD graphene, due to a formation of 3D plasmonic nanostructure, which further demonstrates that these plasmon-driven catalytic reactions may be co-catalytic by plasmon and graphene. The theoretical analysis of that will be in detail given in the discussion section. In addition, the SERS peaks of DMAB productions on three substrates are varied with the substrates while the D peak at 1335 cm^−1^ of the graphene also show different intensities. Therefore, to clarify the efficiency of various catalysts, a comparison between DMAB peaks and graphene D peaks was made for 3D graphene substrate and 2D graphene substrate in the Fig. 4c. The results of comparing the peaks of DMAB production at 1396 and 1432 cm^−1^ with the D peak of graphene at 1335 cm^−1^ are I_2D(1396)_/I_2D(1335)_ = 1.66, I_2D(1432)_/I_2D(1335)_ = 2.11, I_3D(1396)_/I_3D(1335)_ = 3.10, I_3D(1432)_/I_3D(1335)_ = 4.24. More than twice enhancement in the relative intensity ratio reveals that the 3D structure of graphene/Ag-nanoparticles is much more efficient for enhancing plasmon-driven catalytic reactions than 2D plane structure.

Figure 4a demonstrates the molecular limitation concentration dependent graphene enhancing plasmon-driven catalytic reaction at the incident light of 1 mW. It is found that the with the decrease of molecular concentrations, the ratio of Raman intensity at 1432 cm^−1^ over 1335 cm^−1^ is gradually decreased. Even at 10^−11^ M, the Raman profile is at 1432 cm^−1^ of DMAB can be observed in Fig. 4b, which means that the graphene enhancing plasmon-driven catalytic reaction still occurs, even at very low concentration of 10^−11^ M. This result is much better than our previous plasmon (alone)-driven catalytic reaction at the molecular concentration 10^−7^ 1,3. To demonstrate the uniformity and efficiency at large areas on the substrate in Fig. 1d, we measured the Raman spectra (concentration 10^−5^ M) at 10×10 μm. The ratio of Raman intensities of DMAB at 1432 cm^−1^ over Raman intensities of graphene at 1335 cm^−1^ can be seen in Fig. 4c. It is found that the ratio is in the range from 2 to 3, and the averaged ratio is 2.5, which indicates that graphene enhancing plasmon-driven catalytic reaction on our substrate is uniform and efficient. In addition, the repeatability of SERS spectra was measured by ten SERS spectra at intervals, as shown Fig. 4d. Taking the SERS spectrum as a reference and subtracting the other nine SERS spectra from it, the differences can be seen from Fig. 4e, which demonstrates that the SERS spectra have a good repeatability. Figure 4f is a subsequent 2D SERS spectra (the same data of SERS spectra in Fig. 4d), which also reveals that the SERS spectra are repeatable across large areas.

**Figure 5:**
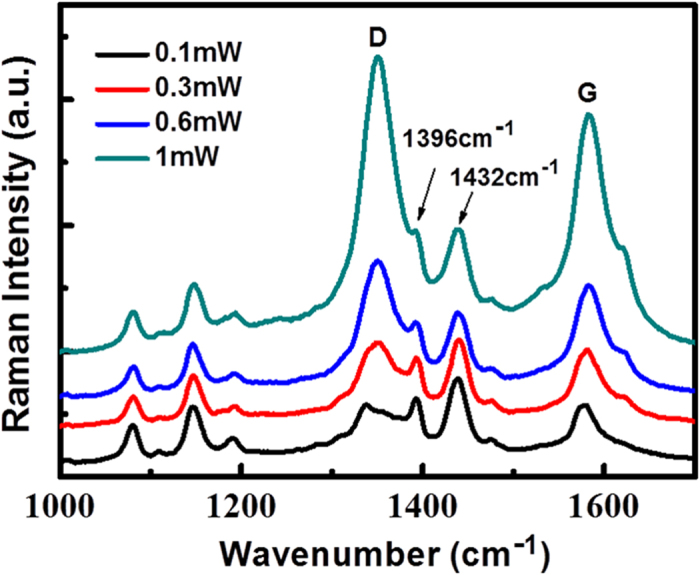
Graphene nanosheets enhancing plasmon-driven reduced reaction, where 4NBT was partially converted to DMAB; which can be compared directly with Fig. 2S(b) to analyze the co-driven reduced reaction process.

It is known that DMAB can also be converted from 4NBT. It is interesting to study the laser power dependent probability and efficiency. Figure 5 demonstrated the plasmon and graphene co-driven catalytic reaction, where 4NBT was partially reduced to DMAB. The Raman spectrum of 4NBT and graphene can be compared with that of DMAB, as shown in Fig. 2S (b). From a direct comparison, we can find that the reduced catalytic reaction (from 4NBT to DMAB) is a partial catalytic reaction at 0.1 mW, since the Raman peak of stretching mode of NO_2_ at 1361 cm^−1^ can also be found. By increasing of laser power, Raman peaks of the graphene are significantly enhanced, but the Raman peak of DMAB at 1432 cm^−1^ only increases a little along with the increase of laser power. By increasing the laser intensity, it seems that the probability of such catalytic reaction is increased slightly. So, it seems that the reduced catalytic reaction is not as good as the oxidized catalytic reaction (from PAPT to DMAB) in this 3D hybrids nanostructure, and the reason for this will be discussed in the next section.

### Mechanism of Plasmon-driven Catalytic Reactions Enhanced by Graphene-Nanoparticle Hybrids

**Figure 6:**
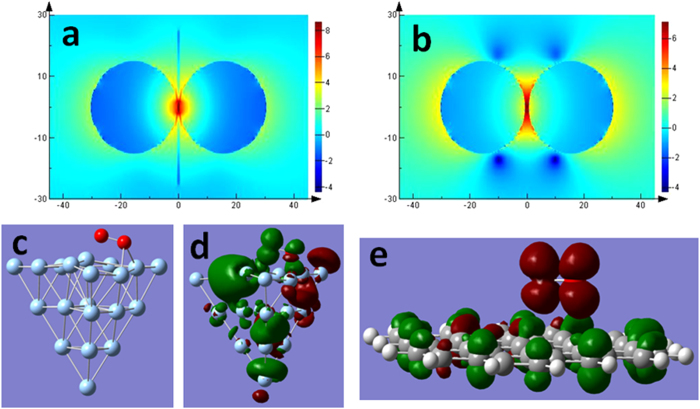
Mechanism of graphene enhancing plasmon-driven catalytic reaction. (**a,b**) show the electric field distribution of a nanoparticle dimer with and without graphene, (**c**) is the structure of O_2_ adsorbed on Ag cluster, (**d**) portrays the electron transfer from O_2_ to Ag cluster on the triplet electronic excitation at 533 nm, (**e**) is the charge transfer upon the excitation of singlet oxygen at ground state to the triplet excited state, where the green and red stand for holes and electrons, respectively.

The contribution of graphene nanosheet sandwiched by Ag nanoparticles (see Fig. 1d) to the plasmon and graphene co-driven catalytic reactions can be firstly estimated with the finite difference time domain (FDTD) method. Our theoretical results reveal that there are stronger EM enhancements for the structure of graphene sandwiched by Ag nanoparticles compared to the nanoparticle dimer, as shown in Fig. 6a,b. The reason may be that graphene provides additional electrons for collective electron oscillations of the surface plasmon. In other words, the collective electron oscillations of the surface plasmon across the graphene can temporarily provide more electrons above the Dirac point of graphene. So, the graphene can increase plasmonic intensity and harvest more energy in this nanogap.

It has also been shown that surface plasmon resonance can activate the oxygen to the triplet excited state, which plays very important role in plasmon-driven catalytic reactions^10^. Our theoretical calculations reveal that one O atom of one molecule of singlet O_2_ adsorbs on two Ag atoms of a cluster at the ground state, as shown Fig. 6c. Excited by 533 nm, the electrons can be transferred from O_2_ to the Ag cluster on the triplet electronic transition of O_2_ adsorbed on the Ag cluster, as displayed in Fig. 6d. The next contribution of graphene on plasmon-driven catalytic reaction is to oxidize singlet ground oxygen adsorbed on graphene to triplet oxygen, in which process the electrons of graphene are transferred to triplet-excited oxygen (see Fig. 6e). So the graphene provides electrons to triplet excited O_2_, and the electrons on triplet excited O_2_ transfer to the Ag cluster. This is a very good process for the plasmon-driven catalytic reaction. The lowest energy of transition (*S*_1_) from singlet oxygen at ground state to the triplet excited oxygen is 1.7726 eV. The *S*_2_ transition (2.2234 eV) can also result in the triplet excited state for oxygen, since the related energy is also lower than the laser frequency (532 nm) that we used. So, it is occurred that plasmon and graphene co-catalytic surface catalytic reactions in our experiments, in which graphene play a key role in enhancing plasmon-driven catalytic reactions.

It has been found that the reduced catalytic reaction (from 4NBT to DMAB) is not as good as the oxidized catalytic reaction (from PAPT to DMAB). The reason is that in the oxidized reaction, the hot electrons generated from plasmon decay play an important role only in decreasing the reaction barrier, where the hole electrons temperately attach to the molecules, and then the neutral potential energy surface (PES) being ion PES^1^,^2^,^3^,^4^,^5^,^6^,^7^,^8^,^9^,^10^. While for the reduced reaction, the other important role is that hot electrons have to provide for catalytic reactions^11^,^12^,^13^,^14^,^15^,^16^. For the reduced catalytic reaction, four electrons are needed for producing one DMAB from two 4NBT^11^,^12^,^13^,^14^,^15^,^16^. So, this is one important reason that the probability of plasmon and graphene co-driven oxidized reaction is better than that of plasmon and graphene co-driven reduced reaction. The second reason is that the potential energy surface of 4NBT is lower than that of PATP, and the potential barrier of 4NBT converting to DMAB that needs to be overcome is larger than that of PATP converting to DMAB^19^.

In summary, we designed and fabricated a 3D plasmonic hybrid nanostructure of flower-like graphene nanosheets sandwiched by Ag nanoparticles on silicon nanocone arrays, which has been applied for graphene enhancing plasmon-driven catalytic reactions and showed the more superiority than 2D graphene-nanoparticles hybrids. Our experimental results reveal that the sensitivity of the co-driven catalytic reaction in this 3D plasmonic hybrid nanostructure can be improved by fourfold over that of the plasmon-driven catalytic reaction. The mechanism of plasmon and graphene co-driven catalytic reaction has been theoretically revealed for the first time. Our results are very valuable for designing and fabricating excellent SERS substrates based on graphene and plasmon co-driven surface catalytic reactions, graphene-based surface plasmon sensors, and so on.

### Experimental section

The silicon nanocone arrays are fabricated by the maskless etching in the inductively coupled plasma (ICP) system, where O_2_ and SF_6_ are used as the etching gas to fabricate the Si nanocones. Geometry of Si nanocones, for example, the height, and density as well as the aspect ratio, were modulated through changing etching parameters. Samples of the Si nanocone array were fabricated at the conditions: SF_6_/O_2_ = 22/7, pressure of 6 mTorr, ICP power of 800 W, and the etching time of 7 minutes. An as-formed silicon nanocone array served as the substrate to grow the few-layer graphene nanosheets by hot filament chemical vapor deposition (HFCVD). The Ar, CH_4_ and H_2_ were fed into the HFCVD system with the ratio of 40:10:1 for the growth of the petaloid graphene nanosheets (PGNSs). And, the growth processes were carried out at the temperatures of 1000 °C, where the pressure was maintained at 2 kPa. The positive bias voltage was added on the filament during growth. The bias current was measured with the ammeter. The structure, morphology and the chemical bond-state of the obtained samples were characterized by the scanning electron microscopy (SEM), and transmission electron microscopy (TEM), and high resolution transmission electron microscopy (HRTEM) (at 200 kV), the electron energy loss spectroscopy (EELS), and the Raman spectra, respectively. And then, the Ag nanospheres were prepared by the 6 nm thick Ag nanofilm deposited on the PGNSs using the E-beam evaporation.

SERS measurements were performed with 532 nm laser with the different powers from 0.1 mW to 1 mW. The objective lens is used to focus the excitation laser on the samples, and to collect Raman spectra with the 1μm laser spot. The as-fabricated graphene nanosheets/Ag-nanoparticles hybrids structure substrates are immersed in 10^−5^ M PATP or 4NBT ethanol solution for 4 hours, and then which was rinsed with the ethanol to get rid of the non-adsorbed molecules. The 10^−5^ M solution was gradually diluted to 10^−6^ M and up to 10^−11^ M by Multiple dilutions.

FDTD was used to demonstrate the plasmon intensity and field distribution in our substrate, which revealed the contribution of graphene to electronic field enhancement. The diameter of Ag nanoparticles is set at 30 nm and the graphene sheet is set at 1 nm (3 layers). Fermi energy, mobility and Fermi velocity of graphene in the calculation are 0.4 eV, 1500 cm^2^/(V*s) and 1×10^6^ m/s, respectively. The ground state geometry of graphene-O_2_ system was optimized with density functional theory (DFT)^37^, and PW91PW91 functional and the 6-31 G(D) basis set. The electronic transition from singlet ground state oxygen adsorbed on the graphene to the triplet excited oxygen were calculated with the time dependent DFT (TD-DFT)^38^, PW91PW91 functional and the 6-31 G(d) basis set. All the theoretical calculations were performed with the Gaussian 09 software.

## Additional Information

**How to cite this article**: Zhao, J. *et al.* Three Dimensional Hybrids of Vertical Graphene-nanosheet Sandwiched by Ag-nanoparticles for Enhanced Surface Selectively Catalytic Reactions. *Sci. Rep.* **5**, 16019; doi: 10.1038/srep16019 (2015).

## Supplementary Material

Supplementary Information
