# Corrigendum: Three Dimensional Hybrids of Vertical Graphene-nanosheet Sandwiched by Ag-nanoparticles for Enhanced Surface Selectively Catalytic Reactions

**DOI:** 10.1038/srep17239

**Published:** 2016-01-07

**Authors:** Jing Zhao, Mengtao Sun, Zhe Liu, Baogang Quan, Changzhi Gu, Junjie Li

Scientific Reports 5: Article number: 1601910.1038/srep16019; published online: 11022015; updated: 01072016.

The original version of this Article contained a typographical error in the spelling of the author Mengtao Sun, which was incorrectly given as Mentao Sun. This has now been corrected in the PDF and HTML versions of the Article.

