# Supplementary material for: Three Dimensional Hybrids of Vertical Graphene-nanosheet Sandwiched by Ag-nanoparticles for Enhanced Surface Selectively Catalytic Reactions
Source: Sci Rep. 2015 Nov 2;5:16019. doi: 10.1038/srep16019 (PMC4629187; doi:10.1038/srep16019)
Supplement: Supplementary Information [file srep16019-s1.pdf]

Supporting Information:

Three Dimensional Hybrids of Vertical Graphene-nanosheet Sandwiched by Ag-nanoparticles for Enhanced Surface Selectively Catalytic Reactions

Jing Zhao,[+] Mentao Sun,[*,+] Zhe Liu, Baogang Quan, Changzhi Gu, [*] Junjie Li[*]

Beijing National Laboratory for Condensed Matter Physics, Institute of Physics, Chinese Academy of Sciences, P. O. Box 603, Beijing 100190, China

[*] E-mail: mtsun@iphy.ac.cn (M.Sun), jjli@iphy.ac.cn (J. Li) and czgu@iphy.ac.cn (C. Gu).

[+] Contributed Equally.

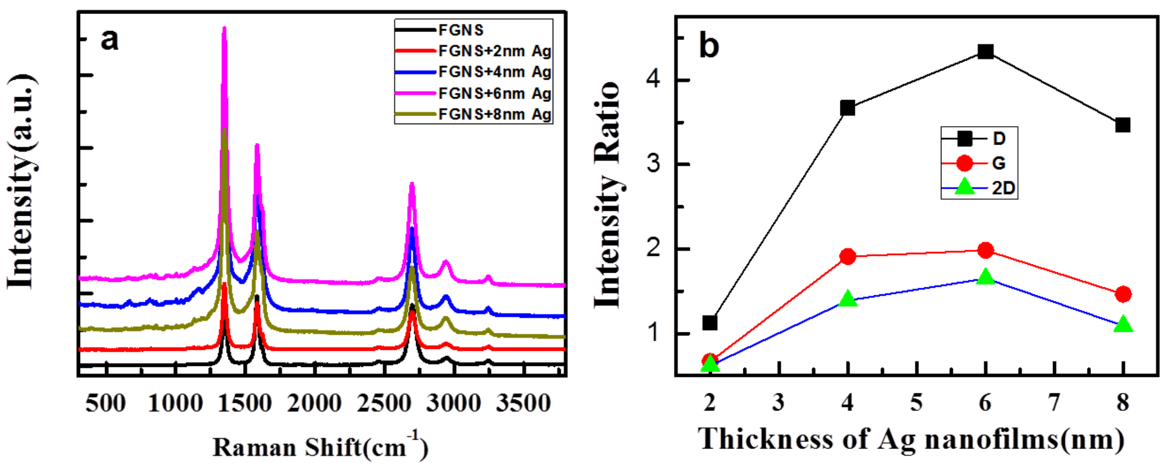

Figure 1S | (a) The SERS spectra of 3D hybrid nanostructures of FGNS/Ag nanofilms with different thickness from 2 to 8nm deposited on both sides of graphene nanosheet, (b) The intensity ratio of the D, G and 2D peak of FGNSs with different thick Ag nanofilms and without Ag nanofilms. The results indicated that the 6nm thick Ag nanofilm coated on graphene nanosheet had the best SERS ability.

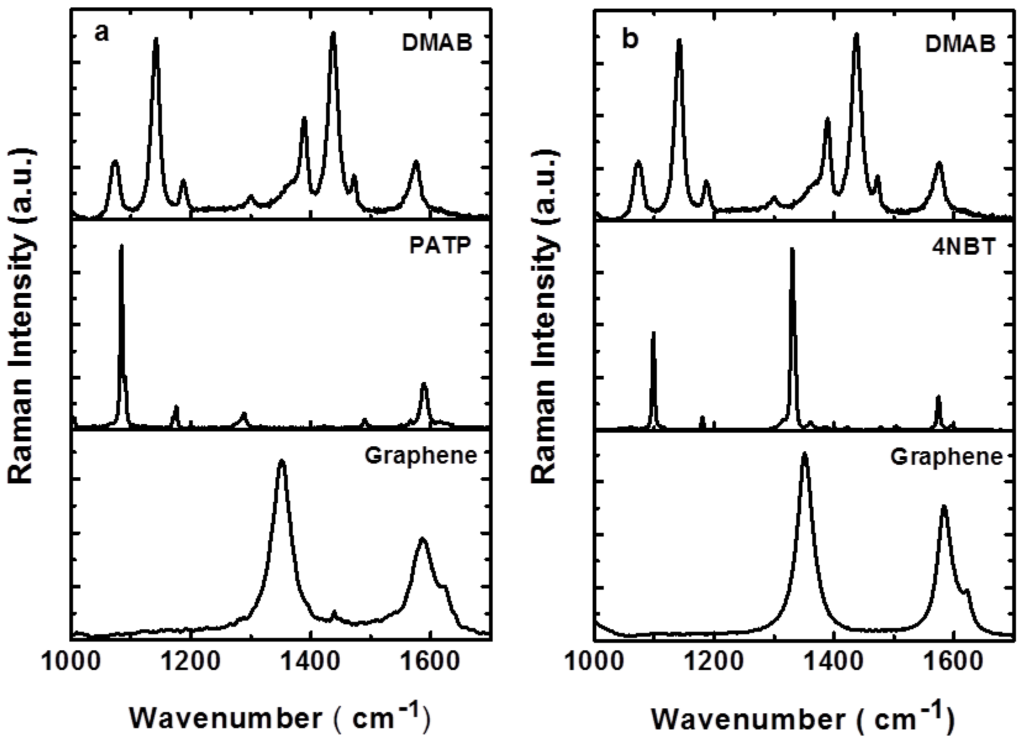

Figure 2S | (a) Normal Raman spectrum of DMAB, PATP and graphene. (b) Normal Raman spectrum of DMAB, 4NBT and graphene.
